# Adherence and persistence with istradefylline treatment in patients with Parkinson’s disease in the United States

**DOI:** 10.3389/fphar.2025.1548631

**Published:** 2025-05-06

**Authors:** Reversa Joseph, Joyce Qian, Hannah Cummings, Yen-Hua Chen, Shubham Tewari, Michael Soileau

**Affiliations:** ^1^ Chalmers P. Wylie Ambulatory Care Center, Columbus, OH, United States; ^2^ Kyowa Kirin, Inc., Princeton, NJ, United States; ^3^ KMK Consulting Inc., Morristown, NJ, United States; ^4^ Texas Movement Disorder Specialists, PLLC., Georgetown, TX, United States

**Keywords:** Parkinson’s disease, adherence, persistence, istradefylline, treatment patterns

## Abstract

**Background:**

Medication adherence and persistence are associated with better outcomes for patients with Parkinson’s disease.

**Objectives:**

To evaluate 12-month adherence and persistence with istradefylline and identify factors associated with persistence among patients.

**Methods:**

A retrospective cohort study was conducted using IQVIA longitudinal prescription (LRx) and medical (Dx) claims data. Adult patients initiating istradefylline between 2019 and 2022 with ≥1 LRx and ≥1 Dx claim every 6 months over 12-month baseline and follow-up periods were included. Adherence was measured by the proportion of days covered (PDC) and medication possession ratio (MPR). Persistence was measured by the duration of prescription fills, allowing for a ≤60-day gap. Multivariate logistic regression was used to evaluate factors associated with 12-month persistence.

**Results:**

Among 2,045 patients, 76.0% were covered by Medicare Advantage and 23.9% were commercially insured. Over 12 months of follow-up, median adherence (both PDC and MPR) was 74.0% and median persistence was 276 days. In a subgroup analysis, median adherence and persistence were significantly greater in patients with Medicare Advantage *versus* commercial insurance (PDC and MPR: 82.2% vs. 49.3%; persistence: 365 days vs. 180 days; all P < 0.001). Treatment initiated at 40 mg *versus* 20 mg (odds ratio [OR]: 1.33 [95% confidence interval [CI]: 1.10, 1.62]) and Medicare Advantage *versus* commercial insurance (OR: 1.79 [95% CI: 1.42, 2.26]) were independently associated with longer persistence.

**Conclusion:**

Patients initiating istradefylline at 40 mg *versus* 20 mg and those with Medicare Advantage *versus* commercial insurance were more likely to have higher adherence and longer persistence with istradefylline.

## 1 Introduction

Parkinson’s disease (PD), a progressive, degenerative disorder characterized by motor and non-motor symptoms, is estimated to affect between 6 and 8.5 million people worldwide (Armstrong and Okun, 2020; American Academy of Neurology Institute, 2021; World Health Organization, 2023; Hindle, 2010; National Institute of Neurological Disorders and Stroke, 2023). In the United States (US), cases of PD are projected to rise to more than 1.2 million by 2030 as the population ages (American Academy of Neurology, 2021; Marras et al., 2018; United States Census Bureau, 2020).

The “gold standard” treatment option for the motor symptoms of PD is levodopa, typically formulated as carbidopa–levodopa (American Academy of Neurology, 2021; Schade et al., 2020; Cummins and Cates, 2022; Schapira, 2007; Grimes et al., 2019; National Institute for Health and Care Excellence, 2017). Although levodopa can provide robust symptom control it cannot stop disease progression and eventually becomes less effective in most patients, leading to motor symptom fluctuations and dyskinesia (Armstrong and Okun, 2020; National Institute of Neurological Disorders and Stroke, 2023; Schapira, 2007; Berger et al., 2020; Isaacson et al., 2022a; Isaacson et al., 2022b). Patients consequently experience “OFF” episodes between medication doses, when worsening of symptoms and functional impairment occur (Armstrong and Okun, 2020; Chou et al., 2018). After 5 years of levodopa therapy, up to 50% of patients will experience “OFF” episodes, increasing to almost 100% after 10 years (Isaacson et al., 2022a; Perepezko et al., 2020; Kalia and Lang, 2015). As PD progresses, patients often require increased dosages of levodopa and the use of additional medications to manage symptoms. Adjunctive therapies to levodopa that target the dopaminergic system, including dopamine agonists, COMT (catechol-*O*-methyl transferase) inhibitors, and MAO-B (monoamine oxidase-B) inhibitors, may be added to the treatment regimen of patients to help control “OFF” episodes (American Academy of Neurology, 2021; Schapira, 2007; National Institute for Health and Care Excellence, 2017; Berger et al., 2020; Isaacson et al., 2022a).

Istradefylline is the first non-dopaminergic adjunctive therapy to carbidopa–levodopa that specifically targets the adenosine A2A pathway. While levodopa and other dopaminergic therapies restore dopaminergic stimulation of the indirect GABAergic striatal output pathway to reverse the enhanced GABAergic tone to the external globus pallidus (GPe) and thereby improve motor function, this effect is opposed by the excitatory effects of adenosine, which stimulates the A2A receptor to increase GABAergic transmission. By comparison, as an adenosine A2A antagonist istradefylline is believed to block adenosine A2A receptors in the striatum and GPe, thereby reducing the excessive output of the indirect striatal output pathway of the basal ganglia and improving motor control by removing a “brake” from the indirect pathway (Jenner et al., 2021; Hauser et al., 2021). The treatment, which is indicated as adjunctive therapy to carbidopa–levodopa for adult patients with PD who experience “OFF” episodes between medication doses, was approved in the US in 2019 (Armstrong and Okun, 2020; Chou et al., 2018; NOURIANZ, 2023). Across multiple clinical trials and real-world studies, istradefylline was well tolerated and demonstrated a sustained reduction in “OFF” time (National Institute for Health and Care Excellence, 2017; Hauser et al., 2021; LeWitt et al., 2008; Mizuno et al., 2010; Kondo et al., 2015; Di Luca et al., 2022; Takahashi et al., 2022; Hatano et al., 2024; Hattori et al., 2023).

Medication non-compliance has been reported to be between 10% and 67% in patients with PD (Malek and Grosset, 2015; Davis et al., 2010; Richy et al., 2013; Tarrants et al., 2010). As PD is a chronic, progressive disease that requires ongoing pharmacological treatment, the burden of increasingly complex medication regimens tends to affect treatment compliance, as well as quality of life (Malek and Grosset, 2015; Castro et al., 2021; Soileau et al., 2023; Zipprich et al., 2021; Wei et al., 2013). Adherence and persistence with PD medications have been shown to be associated with better patient outcomes and a lower healthcare burden (Malek and Grosset, 2015; Davis et al., 2010; Richy et al., 2013; Castro et al., 2021; Shoval et al., 2017). Patients with PD who are compliant with therapy are more likely to achieve the treatment goals of improved motor and non-motor functions, have slower disease progression, and experience improved quality of life (Straka et al., 2019; Grosset et al., 2009). Thus, it is important to understand whether suboptimal outcomes in patients with PD reflect the effectiveness of a given treatment regimen or might be attributable to poor treatment compliance (Tarrants et al., 2010). Insights into treatment compliance with newer medications such as istradefylline that have distinct mechanisms of action would therefore be valuable for clinical decision-making.

To date, no study has examined treatment adherence and persistence with istradefylline in real-world clinical practice. This study was performed to understand the real-world utilization patterns of istradefylline among patients with PD in the US and to assess adherence and persistence with treatment, including an examination of potential factors associated with persistence.

## 2 Materials and methods

### 2.1 Study design

This was a US retrospective cohort study based on de-identified, patient-level administrative claims data from the longitudinal IQVIA Anonymized Patient Level Database (APLD). Medical data in the APLD are drawn from approximately 1 billion outpatient medical claims per year and pharmacy data consist of claims covering approximately 90% of all dispensed prescriptions from US retail pharmacies.

Adults (≥18 years) with PD who initiated istradefylline treatment between September 1, 2019 and March 31, 2022 were included in the study. Patients were required to have ≥1 medical claim and ≥1 pharmacy claim every 6 months over 12-month baseline and follow-up periods. The diagnosis of PD was verified by having ≥1 medical claim with an International Classification of Diseases 10th Revision, Clinical Modification (ICD-10-CM) diagnosis code of G20.x, G21.x during the 12-month baseline period. Patients with evidence of clinical trial participation (ICD-10-CM of Z00.6) during the study period were excluded.

Patients were evaluated overall. In addition, select comparisons were made between subgroups of patients with Medicare Advantage *versus* commercial insurance.

### 2.2 Outcomes and measures

Patient characteristics were evaluated in the baseline period and included age, gender, geographic region, and insurance type, as well as Charlson Comorbidity Index (CCI; Quan adaption) (Quan et al., 2005; Quan et al., 2011) scores, dose of levodopa-containing medications, levodopa equivalent daily dose (LEDD), and the presence of common PD-related conditions. The LEDD is an artificial calculation of the total levodopa dose a patient is receiving daily across multiple PD treatments. LEDD is frequently used in research and clinical practice settings as a standardized method for comparing different PD medication regimens (Schade et al., 2020; Tomlinson et al., 2010; Verber et al., 2020; Nyholm and Jost, 2021; Julien et al., 2021).

Istradefylline utilization and treatment adherence and persistence were evaluated over the 12-month follow-up period. Adherence was measured using two methods: the proportion of days covered (PDC), calculated as the percentage of days covered by filled istradefylline prescriptions over 12 months; and medication possession ratio (MPR), calculated as the percentage of days of supply of istradefylline prescriptions over 12 months, which credits overlapped prescriptions. Persistence was measured as the duration of time a patient continued to fill istradefylline prescriptions over 12 months, allowing for a 60-day grace period (i.e., gap of ≤60 days).

Additionally, a risk factor analysis was conducted to explore potential variables associated with 12-month persistence with istradefylline treatment.

### 2.3 Statistical analyses

Continuous variables were summarized with mean, standard deviation (SD), median, interquartile range (IQR), and minimum and maximum values. Categorical variables were described with counts and percentages. Subgroup comparisons were performed using the Wilcoxon signed-rank test. Kaplan–Meier (KM) curves were used to assess 12-month persistence, as determined by the time to treatment discontinuation.

Multivariate logistic regression was used to evaluate factors associated with 12-month persistence, including patient demographics, CCI, istradefylline initial dose, use of PD adjunctive medications by class, number of PD medications, levodopa (carbidopa–levodopa, carbidopa–levodopa–entacapone, or levodopa inhalation powder) dose, LEDD, and presence of PD-related conditions.

## 3 Results

### 3.1 Sample selection and patient characteristics

**FIGURE 1:**
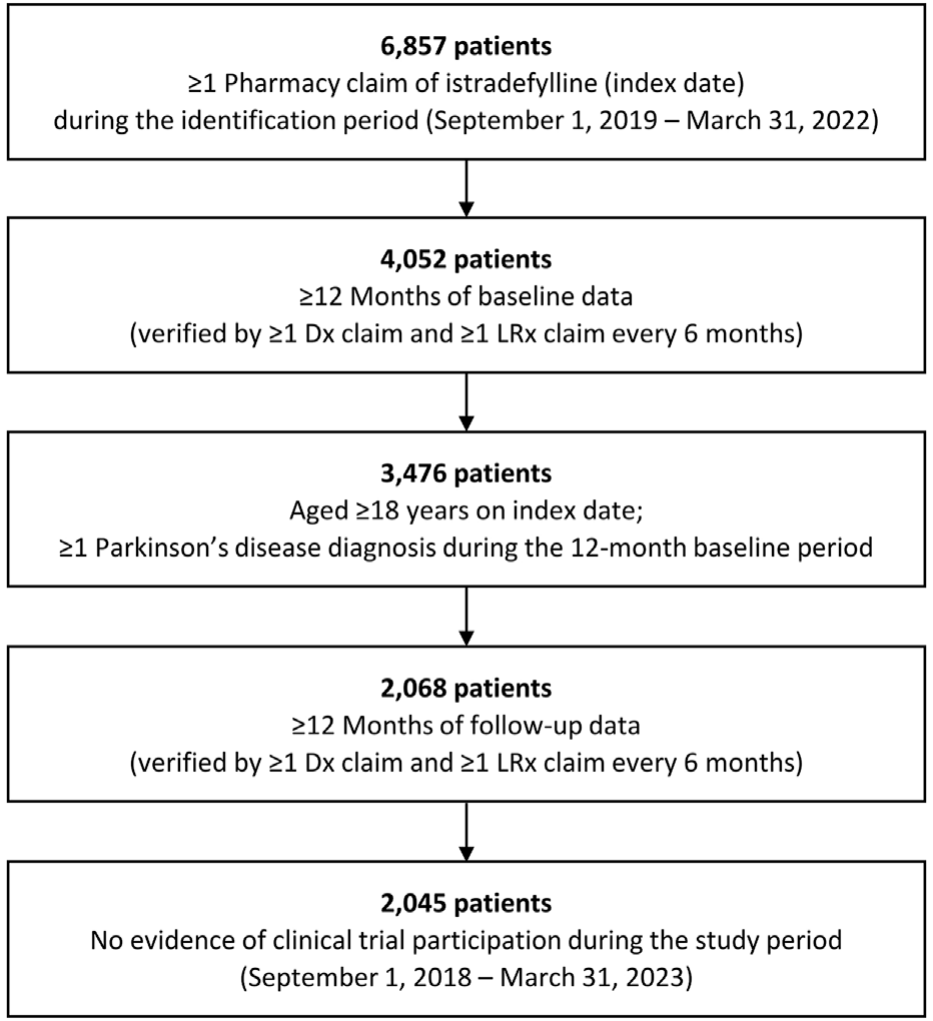
Selection for study cohort of patients with Parkinson’s disease with istradefylline prescription fills. Dx: medical; LRx: prescription.

Overall, 2,045 patients who initiated istradefylline from September 1, 2019 to March 31, 2022 were identified and included in the study (Figure 1), including 1,554 (76.0%) patients categorized as having Medicare Advantage healthcare coverage and 488 (23.9%) categorized as commercially insured (Table 1). As only two patients (0.1%) in the study had other insurance coverage (Medicaid), they were included within the Medicare Advantage group (three patients classified as self-pay were not included in the subgroup analyses).

**TABLE 1:** Baseline characteristics of patients with Parkinson’s disease with istradefylline prescription fills, overall and by insurance status.

	All patients N = 2,045	Medicare advantage^a^ n = 1,554	Commercial n = 488
Age group, n (%)			
18–64 years	319 (15.6)	129 (8.3)	190 (38.9)
65–74 years	765 (37.4)	602 (38.7)	161 (33.0)
75+ years	961 (47.0)	823 (53.0)	137 (28.0)
Age (years)			
Mean (SD)	72.9 (8.6)	74.6 (7.4)	67.7 (9.9)
Median (IQR)	74.0 (68.0–80.0)	75.0 (70.0–80.0)	66.0 (61.0–75.5)
Min-Max	27.0–85.0	32.0–85.0	27.0–85.0
Gender, n (%)			
Female	805 (39.4)	626 (40.3)	178 (36.5)
Male	1,240 (60.6)	928 (59.7)	310 (63.5)
Geographic region, n (%)			
Northeast	552 (27.0)	431 (27.7)	121 (24.8)
Midwest	273 (13.3)	211 (13.6)	62 (12.7)
South	900 (44.0)	663 (42.7)	234 (48.0)
West	312 (15.3)	243 (15.6)	69 (14.1)
Other or missing	8 (0.3)	6 (0.4)	2 (0.4)
Insurance type, n (%)			
Medicare Part D	1,552 (75.9)	1,552 (99.9)	0 (0.0)
Commercial	488 (23.9)	0 (0.0)	488 (100.0)
Cash	3 (0.1)	0 (0.0)	0 (0.0)
Medicaid	2 (0.1)	2 (0.1)	0 (0.0)
CCI score			
Mean (SD)	1.0 (1.5)	1.1 (1.6)	0.8 (1.3)
Median (IQR)	0 (0–2)	0 (0–2)	0 (0–2)
Min-Max	0–11	0–11	0–7
PD-related conditions, n (%)			
Anxiety	487 (23.8)	372 (23.9)	115 (23.6)
Cognitive impairment	127 (6.2)	103 (6.6)	23 (4.7)
Depression	443 (21.7)	352 (22.7)	90 (18.4)
Falls/fractures	349 (17.1)	286 (18.4)	61 (12.5)
Fatigue	487 (23.8)	377 (24.3)	107 (21.9)
Gait abnormalities	496 (24.3)	402 (25.9)	91 (18.6)
Hallucination	116 (5.7)	89 (5.7)	27 (5.5)
Sleep disorder	561 (27.4)	420 (27.0)	140 (28.7)
Tremor	178 (8.7)	123 (7.9)	55 (11.3)
Levodopa dose (mg/day)^b^ ^,^ ^c^			
Mean (SD)	656.4 (422.3)	N/A	N/A
Median (IQR)	584.5 (335.8–893.8)	N/A	N/A
Min-Max	4.1–2671.9	N/A	N/A
LEDD (mg/day)^c^ ^,^ ^d^			
Mean (SD)	740.5 (541.5)	751.5 (538.8)	706.2 (549.7)
Median (IQR)	665.8 (328.4–1,060.4)	672.3 (345.2–1,066.4)	642.1 (271.6–1,033.5)
Min-Max	0.0–2763.0	0.0–2763.0	0.0–2745.5
Use of PD adjunctive medications, n (%)^e^			
Dopamine agonist	865 (42.3)	651 (31.8)	210 (10.3)
COMT inhibitor	315 (15.4)	228 (11.1)	86 (4.2)
MAO-B inhibitor	574 (28.1)	393 (19.2)	179 (8.8)
Amantadine	396 (19.4)	290 (14.2)	106 (5.2)

^a^ The Medicare Advantage group also included two patients who were Medicaid beneficiaries.

^b^ Levodopa formulations of carbidopa–levodopa (Duopa, Sinemet, Sinemet CR, parcopa, Rytary, Dhivy), carbidopa–levodopa–entacapone (Stalevo), and levodopa inhalation powder (Inbrija).

^c^ Calculated excluding the top 1% outlier values.

^d^ LEDD, was calculated as the sum of LEDD, for each patient in the baseline period based on the methodology and LEDD, conversion factors used in Tomlinson et al., 2010 and Schade et al., 2020 (Schade et al., 2020; Tomlinson et al., 2010).

^e^ Calculated among patients with any PD-related medication (including levodopa) in the baseline period: n = 1,941 of all patients; n = 1,476 patients with Medicare; and n = 460 patients with commercial insurance.

CCI: Charlson Comorbidity Index; COMT: catechol-*O*-methyltransferase; IQR: interquartile range; LEDD: levodopa equivalent daily dose; MAO-B: monoamine oxidase-B; PD: Parkinson’s disease; SD: standard deviation.

The mean age of the study population was 72.9 years and approximately 40% of patients were female. The mean CCI score was 1. PD-related conditions observed in >20% of patients included anxiety, depression, fatigue, gait abnormalities, and sleep disorders. Among all patients, the mean baseline dose of levodopa-containing medications was 656.4 mg/day, while the mean LEDD was 740.5 mg/day. Among patients with any recorded PD-related medication in the baseline period (n = 1,941), the most used PD adjunctive medications were dopamine agonists (42.3% of patients) and MAO-B inhibitors (28.1% of patients).

### 3.2 Istradefylline utilization

**FIGURE 2:**
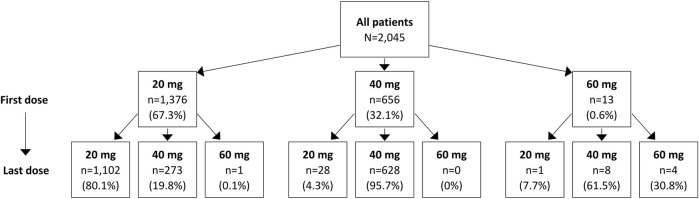
Istradefylline dose change for overall study population of patients with Parkinson’s disease with istradefylline prescription fills. Istradefylline is approved for use at doses of 20 mg and 40 mg. The use of 60 mg is reported to represent real-world experience; the reasons for prescriptions of 60-mg doses could not be determined.

Over the 12-month follow-up period, patients had a median of 5 (IQR: 2–11) istradefylline prescription fills, with a higher number of prescriptions observed among those with Medicare Advantage coverage than commercial insurance (5 [IQR: 2–11] vs. 4 [IQR: 2–10]) (Table 2). Approximately two-thirds of patients (67.3%) initiated istradefylline treatment at a dose of 20 mg. Among patients who initiated istradefylline at 20 mg, 80.1% had prescription fills for 20 mg at 12-month follow-up, while 19.8% had increased their dose to 40 mg (Figure 2). Among patients who initiated istradefylline at 40 mg (32.1% of all patients), 95.7% had prescription fills for 40 mg at 12-month follow-up, while 4.3% had changed to 20 mg.

**TABLE 2:** Istradefylline prescription patterns in the 12 months after patients initiated this treatment, overall and by insurance status.

	All patients N = 2,045	Medicare Advantage^a^ n = 1,554	Commercial n = 488
Initial istradefylline dose, n (%)			
20 mg	1,376 (67.3)	1,047 (67.4)	327 (67.0)
40 mg	656 (32.1)	498 (32.0)	157 (32.2)
60 mg	13 (0.6)	9 (0.6)	4 (0.8)
Year of istradefylline initiation, n (%)			
2019 (Oct–Dec)	9 (0.4)	5 (0.3)	4 (0.8)
2020	372 (18.2)	265 (17.1)	106 (21.7)
2021	1,474 (72.1)	1,136 (73.1)	336 (68.9)
2022 (Jan–Mar)	190 (9.3)	148 (9.5)	42 (8.6)
Treatment duration of istradefylline over 12 months (days)^b^			
Mean (SD)	230.3 (135.5)	239.8 (134.1)	200.6 (135.4)
Median (IQR)	270 (90–365)	300 (90–365)	180 (60–360)
Min-Max	3–365	14–365	3–365
Number of filled istradefylline prescriptions over 12 months, n (%)			
Mean (SD)	6.2 (4.4)	6.4 (4.4)	5.8 (4.5)
Median (IQR)	5 (2–11)	5 (2–11)	4 (2–10)
Min-Max	1–18	1–16	1–18
Istradefylline prescriber type for initial prescription fill, n (%)			
Neurology specialist^c^	1,709 (83.6)	1,286 (82.8)	420 (86.1)
Nurse practitioner	213 (10.4)	168 (10.8)	45 (9.2)
Physician assistant	90 (4.4)	71 (4.6)	19 (3.9)
Other^d^	21 (1.0)	19 (1.2)	2 (0.4)
Missing	12 (0.6)	10 (0.6)	2 (0.4)
Istradefylline prescriber type for all filled prescriptions over 12 months, n (%)			
Neurology specialist^c^	1,793 (80.1)	1,358 (79.3)	432 (82.4)
Nurse practitioner	282 (12.6)	220 (12.9)	62 (11.8)
Physician assistant	115 (5.1)	92 (5.4)	23 (4.4)
Other^d^	49 (2.2)	42 (2.5)	7 (1.3)

Istradefylline is approved for use at doses of 20 mg and 40 mg. The use of 60 mg is reported to represent real-world experience; the reasons for prescriptions of 60-mg doses could not be determined.

^a^ The Medicare Advantage group also included two patients who were Medicaid beneficiaries.

^b^ Treatment duration was calculated as the sum of days of istradefylline supply, capped at 12 months.

^c^ Neurology specialist includes categories of child neurology, clinical neurophysiology, and vascular neurology.

^d^ Other includes dermatology, epilepsy, family medicine, geriatric medicine (internal medicine), hospitalist, internal medicine, nephrology, ophthalmology, pharmacist, physical medicine and rehabilitation, psychiatry, pulmonary disease, and sleep medicine.

IQR: interquartile range; SD: standard deviation.

The mean (SD) duration of istradefylline treatment was 230.3 (135.5) days, which was similar regardless of insurance status (Table 2). Istradefylline was initially prescribed by neurology specialists (including child neurology, clinical neurophysiology, and vascular neurology sub-specialties) in >80% of cases across the study population, and this proportion remained similar across all istradefylline prescriptions during the study period.

### 3.3 Istradefylline adherence and persistence

**FIGURE 3:**
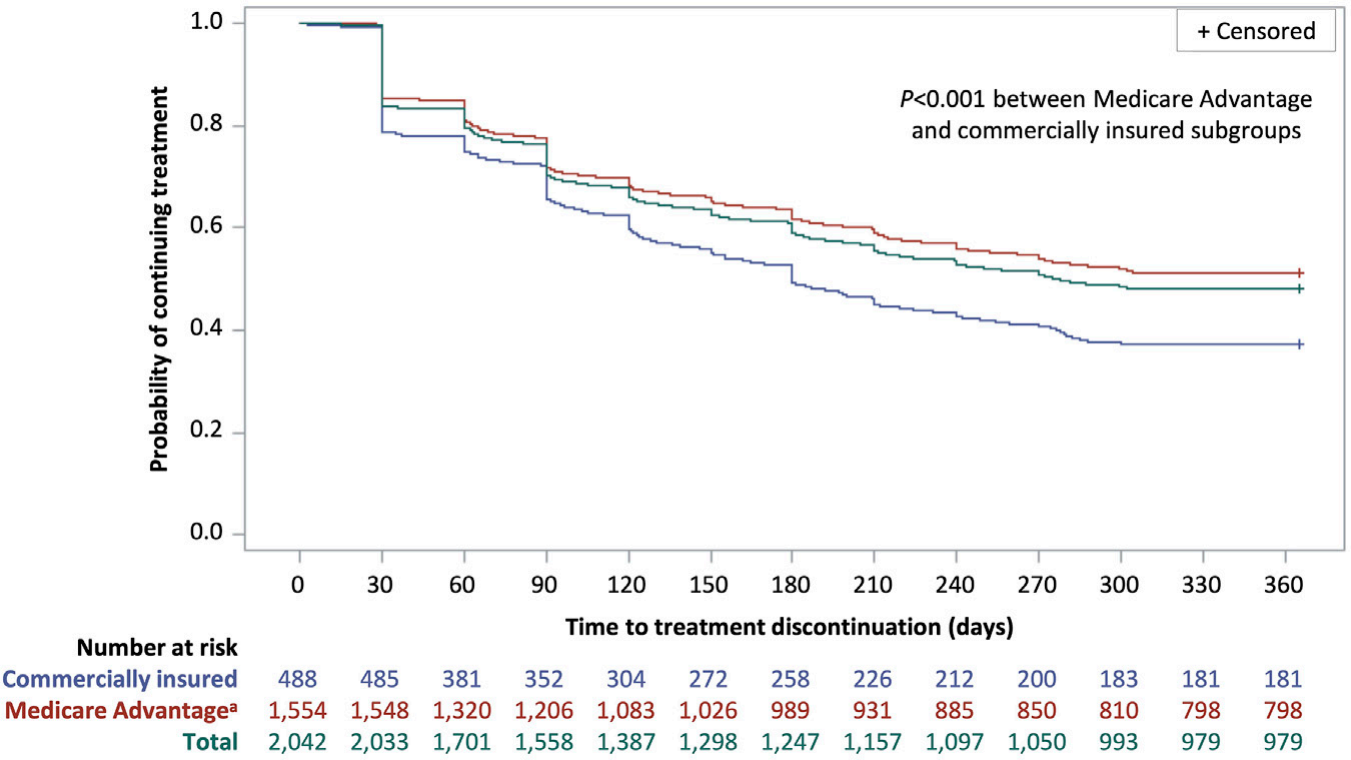
Kaplan–Meier curve of time to treatment discontinuation for patients with Parkinson’s disease with istradefylline prescription fills, overall and by insurance status. Discontinuation was defined as a treatment gap of >60 days between the exhaustion of a prescription supply and the next prescription refill. Patients who did not discontinue treatment were considered to be persistent to treatment. ^a^The Medicare Advantage group also included two patients who were Medicaid beneficiaries.

Over 12 months, the median treatment adherence with istradefylline was 74.0% as measured by both PDC and MPR and the median duration of persistence was 276 days (Table 3). Median adherence was higher and persistence was longer in patients with Medicare Advantage coverage than among those with commercial insurance (PDC: 82.2% vs. 49.3%; MPR: 82.2% vs. 49.3%; persistence: 365 days vs. 180 days; *P* < 0.001 for all comparisons). An adherence rate of at least 80% (PDC or MPR ≥0.8) over 12 months was achieved by almost half of all patients as measured by both PDC (47.1%) and by MPR (46.8%). Approximately 40% of patients had a 12-month adherence rate of less than 50% (PDC <0.5: 41.4%; MPR <0.5: 41.5%). Slightly over half of all patients (52.1%) discontinued treatment over the 12-month follow-up period (Figure 3). Treatment discontinuation rates were lower among patients with Medicare Advantage coverage (48.6%) than those with commercial insurance (62.9%).

**TABLE 3:** Adherence and persistence to istradefylline over 12 months, overall and by insurance status.

	All patients N = 2,045	Medicare Advantage^a^ n = 1,554	Commercial n = 488
Adherence (PDC, %)			
Mean (SD)	61.8 (36.2)	64.3 (35.8)	53.9 (36.1)
Median (IQR)	74.0 (24.7–98.1)	82.2 (24.7–98.6)	49.3 (16.4–95.9)
Min-Max	0.8–100.0	3.8–100.0	0.8–100.0
Adherence (MPR, %)			
Mean (SD)	63.5 (38.1)	66.1 (37.8)	55.2 (37.8)
Median (IQR)	74.0 (24.7–99.2)	82.2 (24.7–99.7)	49.3 (16.4–97.3)
Min-Max	0.8–156.2	3.8–156.2	0.8–141.6
Persistence (days)			
Mean (SD)	231.6 (140.6)	240.6 (139.8)	203.2 (139.6)
Median (IQR)	276 (90–365)	365 (90–365)	180 (60–365)
Min-Max	3–365	14–365	3–365
12-Month persistence, n (%)			
Yes	980 (47.9)	798 (51.4)	181 (37.1)
No	1,065 (52.1)	756 (48.6)	307 (62.9)

PDC, was calculated as the percentage of days covered by filled istradefylline prescriptions over 12 months; MPR, was calculated as the percentage of days of supply of istradefylline prescriptions over 12 months, which credits overlapped prescriptions.

Persistence was measured as the duration of time a patient continued to fill istradefylline prescriptions over 12 months, allowing for a 60-day grace period.

^a^ The Medicare Advantage group also included two patients who were Medicaid beneficiaries.

IQR: interquartile range; MPR: medication possession ratio; PDC: proportion of days covered; SD: standard deviation.

In the multivariate regression analysis, istradefylline initiated at a dose of 40 mg (OR: 1.33 [95% CI: 1.10, 1.62]) and Medicare Advantage coverage (OR: 1.79 [95% CI: 1.42, 2.26]) were both independently associated with longer persistence with treatment in this study (Table 4).

**TABLE 4:** Factors associated with 12-month persistence with istradefylline among patients with Parkinson’s disease.

	Risk Factor	OR (95% CI)
Age group (ref: <65 years)	65–74 years	1.01 (0.76, 1.35)
	75+ years	1.06 (0.79, 1.43)
Gender (ref: male)	Female	0.97 (0.81, 1.18)
Geographic region (ref: West)	Midwest	1.36 (0.97, 1.90)
	Northeast	0.98 (0.73, 1.31)
	South	1.07 (0.82, 1.39)
Insurance type (ref: commercial)	Medicare Advantage	**1.79 (1.42, 2.26)**
CCI score (ref: 0)	1–2	0.98 (0.80, 1.21)
	3+	1.10 (0.83, 1.46)
Istradefylline initial dose (ref: 20 mg)	40 mg	**1.33 (1.10, 1.62)**
Use of PD adjunctive medications (ref: none)	Any PD adjunctive medication	0.84 (0.59, 1.19)
	Dopamine agonist	0.95 (0.68, 1.31)
	COMT inhibitor	0.94 (0.66, 1.33)
	MAO-B inhibitor	1.06 (0.78, 1.45)
	Amantadine	0.84 (0.60, 1.17)
Number of PD medications (ref: 0–1)	2	1.16 (0.81, 1.67)
	3	0.92 (0.55, 1.54)
	4	1.15 (0.58, 2.31)
Levodopa dose^a^ (ref: 0–400 mg)	400–800 mg	0.92 (0.63, 1.34)
	800+ mg	0.87 (0.53, 1.41)
LEDD^b^ (ref: 0–400 mg)	400–800 mg	0.97 (0.66, 1.43)
	800+ mg	0.94 (0.56, 1.59)
PD-related condition (ref: not present)	Anxiety	0.87 (0.70, 1.10)
	Cognitive impairment	1.10 (0.76, 1.60)
	Depression	0.99 (0.78, 1.25)
	Falls/fractures	1.14 (0.90, 1.45)
	Fatigue	0.81 (0.65, 1.01)
	Gait abnormalities	1.09 (0.88, 1.35)
	Hallucination	0.90 (0.61, 1.34)
	Sleep disorder	1.05 (0.85, 1.29)
	Tremor	1.14 (0.82, 1.58)

Factors were evaluated at baseline and upon istradefylline initiation. An OR, with 95% CI, excluding the value of one was considered statistically significant.

^a^ Levodopa formulations of carbidopa–levodopa (Duopa, Sinemet, Sinemet CR, parcopa, Rytary, Dhivy), carbidopa–levodopa–entacapone (Stalevo), and levodopa inhalation powder (Inbrija).

^b^ LEDD, was calculated as the sum of LEDD, for each patient in the baseline period based on the methodology and LEDD, conversion factors used in Tomlinson et al., 2010 and Schade et al., 2020 (Schade et al., 2020; Tomlinson et al., 2010).

CCI: charlson comorbidity index; CI: confidence interval; COMT: catechol-*O*-methyltransferase; LEDD: levodopa-equivalent daily dose; MAO-B: monoamine oxidase-B; OR: odds ratio; PD: Parkinson’s disease; ref: reference group.

## 4 Discussion

This retrospective cohort study provides insights into the real-world use of istradefylline in patients with PD who initiated treatment since the drug’s US approval in 2019. While treatment adherence or persistence with dopaminergic PD medications has been evaluated in previous studies, to our knowledge this study provides the first analysis of adherence and persistence with istradefylline, a non-dopaminergic adjunctive therapy to carbidopa–levodopa.

Among patients who initiated istradefylline at a 20-mg dose, approximately 80% had prescription fills for 20 mg at 12 months, while nearly 20% had increased their dose to 40 mg. By comparison, nearly all patients (96%) who initiated istradefylline at 40 mg had prescription fills for 40 mg at 12 months, with only a small proportion (<5%) lowering their dose to 20 mg. Over 12 months of follow-up from initiation of istradefylline, a median treatment adherence of 74% (using both measures of PDC and MPR) and a median persistence duration of 276 days were observed.

Given that previous studies in PD assessed treatment patterns with dopaminergic agents, comparisons with the current analysis of istradefylline are limited. Nevertheless, in previous studies evaluating treatment adherence with dopaminergic PD medications using commercial and Medicare claims data, rates of adherence by patients were reported to vary between 37% and 73% (Davis et al., 2010; Richy et al., 2013; Tarrants et al., 2010; Wei et al., 2013; Johnsrud et al., 2021; Kulkarni et al., 2008). A more recent study in the Medicaid population reported higher adherence and persistence in patients initiating levodopa (considered the gold standard for treatment of PD) than those initiating a dopamine agonist, but overall <50% of patients in either group were considered adherent (PDC threshold for adherence: ≥0.8) and only approximately 50% of patients initiating levodopa and 40% initiating a dopamine agonist remained persistent with treatment over 12 months (Johnsrud et al., 2021). In another study using commercial and Medicare claims data and conducted prior to the availability of istradefylline, mean adherence (as measured by MPR) following initiation of approved medications for PD was 58% over 12 months, and an adherence rate of at least 80% (MPR ≥0.8) was achieved by <40% of patients (Davis et al., 2010). In this study of istradefylline, median adherence was 74.0% and an adherence rate of at least 80% (PDC or MPR ≥0.8) over 12 months was achieved by almost half of patients, suggesting that adherence with istradefylline is comparable or favorable to adherence with dopaminergic treatments.

Several previous analyses have identified multiple factors associated with treatment adherence in PD. These factors have included age, gender, motor and non-motor symptoms, depressive symptoms, cognition, use of dopamine agonists, number of PD medications, and LEDD exposure (Richy et al., 2013; Tarrants et al., 2010; Castro et al., 2021; Zipprich et al., 2021; Wei et al., 2013; Straka et al., 2019; Johnsrud et al., 2021; Kulkarni et al., 2008; Aggarwal et al., 2021; Daley et al., 2012; Dahodwala et al., 2021; Frazer et al., 2022; Radojević et al., 2022; Yi et al., 2024). For example, in a retrospective US claims data analysis of commercially insured patients, the odds of non-adherence (MPR) was lower in patients <80 years than those ≥80 years and was higher in those with a diagnosis of depression (Richy et al., 2013). In a claims data analysis of patients with Medicare coverage, an LEDD of >1,000 mg/day was associated with lower adherence (PDC) than >800 mg/day (Dahodwala et al., 2021). In addition, in a claims analysis of patients receiving Medicaid, use of levodopa was associated with higher adherence than the use of dopamine agonists (PDC: 62.1% vs. 54.6%) (Johnsrud et al., 2021). Comparisons with such previous studies are limited, however, given that these analyses were conducted in different populations using varying measures of treatment compliance, and also generally evaluated potential factors affecting treatment adherence rather than persistence. As noted, most previous studies also assessed compliance with dopaminergic agents and did not include istradefylline.

A previous retrospective cohort study evaluating treatment adherence with high-dose PD medications in Medicare beneficiaries found that few patients with advanced disease sustained a high-dose medication regimen (LEDD >1,000 mg) over 12 months, but most could sustain a substantially lower-dose regimen (Dahodwala et al., 2021). Real-world analyses have demonstrated a long-term slowing of LEDD escalation in patients who initiated treatment with istradefylline, particularly for those who were receiving higher doses of levodopa (Hattori et al., 2023; Hattori et al., 2022). Patients who initiated istradefylline early and were not receiving concomitant COMT inhibitors or MAO-B inhibitors also showed a reduced escalation in LEDD (Hattori et al., 2023; Hattori et al., 2022). Thus, by contributing to a slowing of LEDD escalation, the use of istradefylline early in the treatment of PD might enable patients to have a lower likelihood of experiencing levodopa-induced complications and thus be more compliant with their PD medication regimens.

Given that a dose change to 40 mg at 12 months of follow-up was observed for almost 20% of patients and the higher likelihood of persistence with a 40-mg initial dose, initiating istradefylline treatment at a 40-mg dose could be preferable to a 20-mg dose to reduce the risk of treatment discontinuation. Istradefylline has been shown to effectively reduce “OFF” time at both 20- and 40-mg doses (Hauser et al., 2021; Takahashi et al., 2022). In a recent analysis, an increase in istradefylline dosing from 20 mg to 40 mg did not substantially increase the incidence of adverse drug reactions, supporting the safety of the 40-mg dose (Takahashi et al., 2022). Unlike dopaminergic agents, the adenosine A2A antagonism of istradefylline acts through a mechanism beyond the dopaminergic system, thereby offering a distinct approach for treating “OFF” time in patents with PD (Jenner et al., 2021). Further research is necessary to evaluate considerations for initial treatment dosing with istradefylline in clinical practice. Additionally, the higher odds of treatment persistence and lower rates of discontinuation observed in our study with Medicare Advantage coverage compared with commercial insurance may be related to reimbursement issues (Fusco et al., 2023; IQVIA Institute for Human Data Science, 2020) and warrant further investigation.

### 4.1 Study limitations

This study had several limitations inherent to its retrospective, real-world study design and use of administrative claims data. The data provide only indirect measures of treatment adherence and persistence and associated risk factors. Pharmacy prescription fills cannot account for whether the medication was taken as prescribed. Additionally, given the nature of claims data, only those disease entities and variables with specific billing codes could be captured. The types of measurable data available were thus limited, including potential risk factors for treatment compliance, such as markers of disease severity. Reasons for treatment discontinuation with istradefylline could also not be determined. As with all claims data, there was also the potential for underestimation of rates of health events and missed events, as well as misclassification due to coding errors. In addition, given that adherence and persistence were evaluated in this study over a 12-month period, further research would be needed to determine longer-term treatment compliance with istradefylline.

Finally, the IQVIA APLD claims database used in this study provides a large, representative sample of patients with Medicare Advantage or commercial insurance coverage in the US. However, the study outcomes may not be generalizable to all patients with PD, such as those with other insurance types.

### 4.2 Conclusions

This real-world US study among 2,045 patients with PD demonstrated a median adherence of 74% and a median persistence duration of 276 days over 12 months. Patients who initiated istradefylline at 40 mg or who had Medicare Advantage healthcare coverage were more likely to have higher adherence and longer persistence over 12 months than those who started dosing at 20 mg or who were commercially insured. Further research into factors associated with adherence and persistence with istradefylline would be beneficial to support treatment decision-making in clinical practice.

## Data Availability

The datasets presented in this article are not readily available because the data were used under license from IQVIA. Data availability is subject to third-party restrictions. Requests to access the datasets should be directed to Joyce Qian, joyce.qian.8y@kyowakirin.com.
